# miR-34a in serum is involved in mild-to-moderate COPD in women exposed to biomass smoke

**DOI:** 10.1186/s12890-019-0977-5

**Published:** 2019-11-27

**Authors:** Yadira Velasco-Torres, Victor Ruiz López, Oliver Pérez-Bautista, Ivette Buendía-Roldan, Alejandra Ramírez-Venegas, Julia Pérez-Ramos, Ramcés Falfán-Valencia, Carlos Ramos, Martha Montaño

**Affiliations:** 1 Department of Biological Systems, Autonomous Metropolitan University-Xochimilco (UAM-X), Mexico City, Mexico; 2 Biological and Health Sciences, Autonomous Metropolitan University-Xochimilco (UAM-X), Mexico City, Mexico; 3 Clinic of Smoking and COPD, Mexico City, Mexico; 4 Laboratory of Molecular Biology, Mexico City, Mexico; 5 Laboratory of Translational Research in Aging and Pulmonary Fibrosis, Mexico City, Mexico; 6 Laboratory of Cell Biology, Department of Research in Pulmonary Fibrosis, Mexico City, Mexico; 7 Laboratory of HLA, National Institute of Respiratory Diseases Ismael Cosio Villegas (INER), Calzada de Tlalpan 4502, Col Section XVI, C.P. 14080, Tlalpan, Mexico City, Mexico

**Keywords:** Biomass smoke exposure, COPD, microRNAs, PCR arrays, RT-qPCR, Tobacco smoking

## Abstract

**Background:**

Chronic obstructive pulmonary disease (COPD) is characterized by persistent respiratory symptoms and airflow limitation that is due to airway and/or alveolar abnormalities. The main causes of COPD are Gene-environment interactions associated with tobacco smoking (COPD-TS) and biomass smoke (COPD-BS). It is well know that microRNAs (miRNAs) participate in the control of post-transcriptional regulation and are involved in COPD-TS; nevertheless, those miRNAS are participating in the COPD-BS are unidentified. Thus, we studied which miRNAs are involved in COPD-BS (GOLD stages I–II).

**Methods:**

In the screening phase, the profile of the miRNAs was analyzed in serum samples (*n* = 3) by means of a PCR array. Subsequently, the miRNAs were validated with RT-qPCR (*n* = 25) in the corresponding study groups. Additionally, the serum concentration of Notch1 was measured comparing COPD-BS vs COPD-TS.

**Results:**

miR-34a was down-regulated in COPD- BS vs COPD-TS. In the other study groups, three miRNAs were differentially expressed: miR-374a was down-regulated in COPD-BS vs C, miR-191-5p was up-regulated in COPD-BS vs H-BS, and miR-21-5p was down-regulated in COPD-TS compared to the C group. Moreover, the serum concentration of Notch1, one of the targets of miR-34a, was increased in COPD-BS compared to women with COPD-TS.

**Conclusions:**

This is the first study in patients with COPD due to biomass that demonstrates miRNA expression differences between patients. The observations support the concept that COPD by biomass has a different phenotype than COPD due to tobacco smoking, which could have important implications for the treatment of these diseases.

## Background

Chronic obstructive pulmonary disease (COPD) is a common, preventable and treatable disease, characterized by persistent respiratory symptoms and airflow limitation. COPD is caused by exposure to noxious particles or gases [1]; tobacco smoke inhalation is a fundamental cause of COPD and affects both genders.

Biomass smoke, such as that produced by wood combustion for cooking, is another risk factor that disproportionally affects women, particularly in low and middle-income countries [2].

Currently, the COPD phenotype by biomass is considered different from that caused by tobacco smoke. Unlike COPD caused by tobacco, biomass COPD tends to remain in GOLD I and II stages [3–6], and rarely progresses to emphysema [5]. Several hypotheses have been proposed to explain the plateau in the development of COPD by biomass. Among them, early airway remodeling is the most accepted explanation, since longitudinal studies have shown a different pattern in airway remodeling in these women. Still, the specific mechanisms that differentiate the phenotype of COPD by tobacco and biomass are largely unknown.

Biomass COPD is usually characterized by chronic bronchitis, persistent cough and phlegm. Recently, the role of miRNAs in the pathophysiology of COPD has been explored, increasing our understanding of their role in the development of phenotypic heterogeneity of COPD. miRNAs could help to the differences in COPD phenotypes; studies in tobacco COPD have reported differential expression of miR-20, miR-28-3p, miR-34c-5p, miR-100 and miR-7 in smokers, ex-smokers and non-smokers.

These miRNAs are involved in cancer detection, protein coding of inflammatory factors, macrophages and vascular inflammation regulators [7–9]. There are not reports regarding to determine the participation of miRNAs in COPD by biomass.

We aimed to compare the expression of microRNAs in women with COPD due to biomass and tobacco smoke, as will as in control women, and to determine and quantify the target of miRNAs that are being differentially expressed by COPD phenotypes.

## Methods

### Study population

**Fig. 1:**
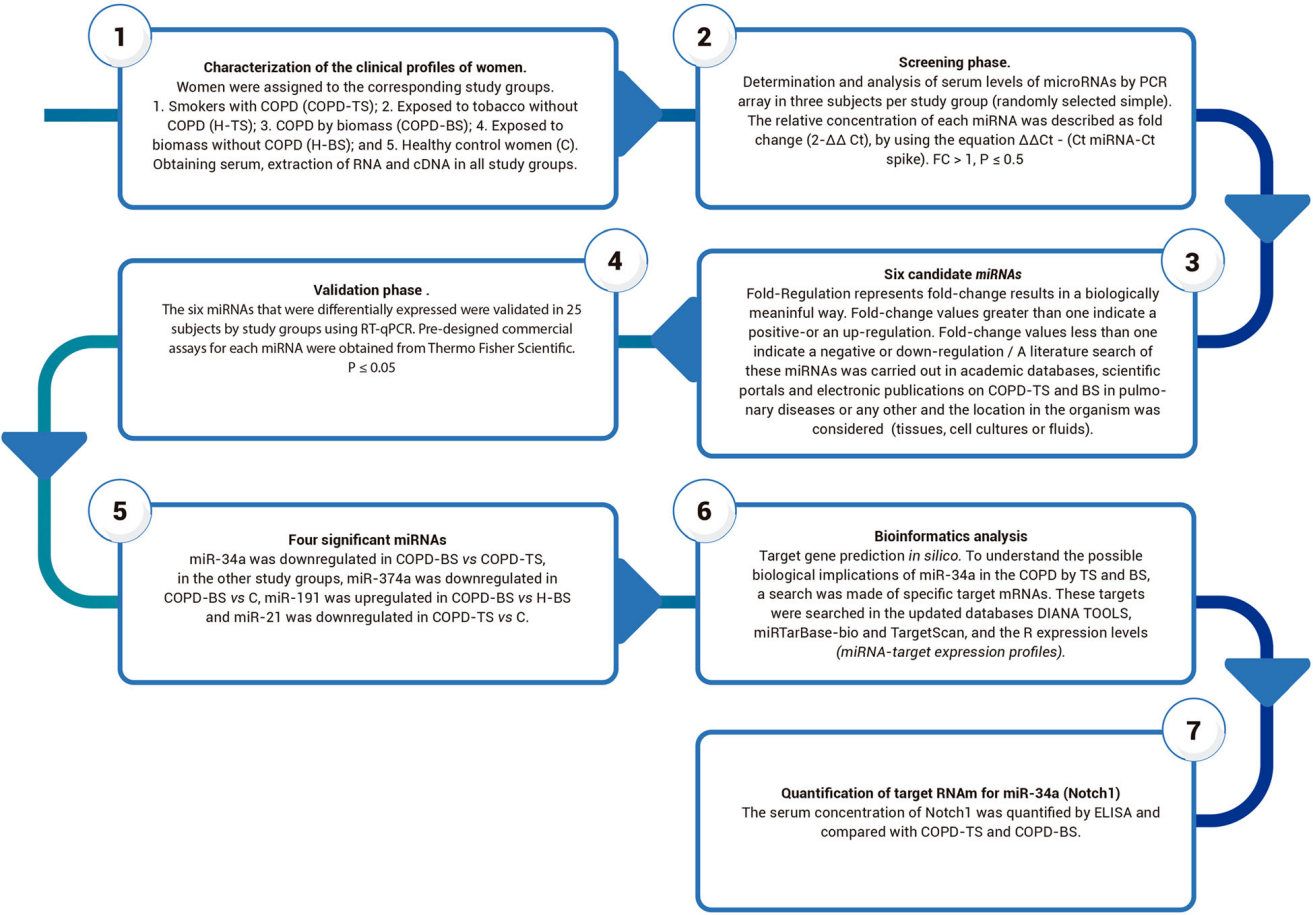
Overview of the study design strategy. Abbreviations: COPD-TS, COPD due to tobacco smoke; COPD-BS, COPD due to biomass smoke. H-BS, women exposed to BS without COPD. COPD-TS, COPD women exposed to TS without COPD. C, control women

A total of 125 women divided in five groups of 25 participants were recruited for the study. We included women with biomass COPD (COPD-BS), tobacco COPD (COPD-TS), smokers without COPD (H-TS), biomass exposed without COPD (H-BS), and healthy female controls (C), whom had not history of exposure to TS or BS, and with absence of any other respiratory or non-respiratory disease as controls (see Fig. 1). The diagnosis of COPD was established according to the history of smoking or exposure to BS and pulmonary function tests followed the recommendations of the American Thoracic Society and European Respiratory Society [1] and using standardized references for Mexican population [10, 11]. All women with COPD had I-II GOLD stages.

Demographic, anthropometric and clinical data were collected including TS history (> 10 packs/year) and cumulative exposure to BS in hours/year by determining the average number of hours/day of exposure and the number of years of exposure; no patient with COPD was exposed to both factors. Wood was the only fuel used by women with COPD-BS, who came from rural and suburban, low-income regions of Mexico.

### Blood samples

Five mL of blood were collected in anticoagulant-free tubes (BD VACUTAINER, Becton, Franklin Lakes, NJ, USA), following the standard procedures at the INER, which included morning only bleeding with at least 8 h fasting. Samples were centrifuged at 5000 g X 15 min and room temperature, to obtain the serum, which was kept at − 20 °C until their analysis.

### Isolation of serum microRNA

The extraction of the miRNAs was performed using the QIAGEN miRNeasy serum/plasma kit (Hilden, Germany) following the manufacturer’s instructions. Aliquots of 200 μL of serum were transferred into 2 mL tubes; QIAzol lysis reagent, 3.5 μL of spike (control), 1.6 × 10^8^ copies/μL, and 200 μL of chloroform were added and subsequently centrifuged at 12,000 g X 15 min at 4 °C. Aqueous phase was separated and 1.5 volumes of 100% ethanol were added; later, an aliquot of 700 μL was passed through a 2 mL RNeasy spin MinElute column and centrifuged at 8000 g X 15 s. Then, 700 μL of Buffer RWT were added to the RNeasy spin MinElute column, which was centrifuged at 8000 g X 15 s followed by the addition of 500 μL of Buffer RPE. The resulting miRNA was eluted with 20 μL of RNase-free water by centrifugation at 10,000 g X 1 min. The miRNA was quantified, and integrity was assessed with the Agilent Bioanalyzer 2100 system (Agilent Technologies, Santa Clara, CA, USA).

### RT-qPCR Array assay in serum

Serum miRNAs measurement was conducted in two stages: a screening stage to identify miRNAs differentially expressed in a small subsample of each participating group and a validation stage to confirm that such miRNAs were indeed different in all participants. In the screening stage we conducted a miRNAs-wide analysis, which included 96 miRNAs, using samples from three randomly selected patients from each study group. Quantitative real-time PCR (RT-qPCR) was used with the miScript miRNA PCR Array Human Serum/Plasma kit from QIAGEN (Hilden, Germany) using the StepOnePlus™ Real-Time PCR System (Applied Biosystems-Real-Time PCR systems Foster City, California, USA). The data analysis was performed using software provided by the manufacturer (available at https://www.qiagen.com/ch/shop/genes-and-pathways/data-analysis-center-overview-page/). Once miRNAs differentially expressed were identified, we implemented the validation stage in the remaining 25 participants of each group. The validation was performed by reverse transcriptase-quantitative polymerase chain reaction (RT-qPCR), obtaining the cDNA of the miRNAs extracted with the RT kit and amplified withTaqMan Universal Master Mix II with the UNG kit, all from Applied Biosystems by Thermo Fisher Scientific (USA). Pre-designed commercial assays for each miRNA were obtained from Thermo Fisher Scientific: hsa-miR-150-5p (Assay ID 000473), hsa-miR-223-3p (Assay ID 0002295), hsa-miR-191-5p (Assay ID 002299), hsa-miR-374a-5p (Assay ID 000563), hsa-miR-21-5p (Assay ID 000397) and hsa-miR-34a-5p (Assay ID 000426). The expression level of each miRNA was evaluated using the comparative threshold cycle method (ΔΔCt) and normalized with a corresponding miRNA sequence from *C. elegans* as an exogenous normalizer in gene expression (*spike-in cel-miR-39*). The relative concentration of each miRNA was described by the equation ΔCt = (Ct miRNA-Ct spike). The cut-off value was set as the cycle ≤40 and it was considered that a gene was not detectable when the Ct was > 40 and the signal was under established limits [12, 13].

### Protein quantification

The serum concentration of the Notch1 protein, whose mRNA is the target of miR-34a, was performed using an ELISA kit (R & D Systems Human Notch1 DuoSet ELISA), following the manufacturer’s instructions.

### Statistical analysis

To obtain the sample size, the free software G Power (version 3.1.9.2; Heinrich-Heine-Universität, Düsseldorf, Germany) was used. According to the results obtained in the screening phase where we found down-regulation of miR-34a, we calculated the sample size from 2 proportions to 30% between patients with COPD due to biomass and COPD due to tobacco.

The demographic and clinical characteristics of the study populations were expressed as mean ± SD. The statistical analysis was carried out by means of ANOVA Tukey’s post-hoc test to multiple comparisons and the differences, while comparison between two groups were determined by Student’s t-test. The statistical analysis for qPCR array was perform with the Qiagen software (available at https://www.qiagen.com/us/shop/genes-and-pathways/data-analysis-center-overview-page/). RT-qPCR was analyzed by relative quantification (ΔΔCt method). The differential expression of a miRNA, and the Notch1 protein quantification was also evaluated by Student’s t-test. The analyses were performed using the statistical package GraphPad version 6.01 (GraphPad Software, Inc., La Jolla, CA, USA). *P* values less than 0.05 were considered significant in all cases.

## Results

### Patient characteristics

Table 1 shows the anthropometric, clinical, and physiological characteristics of the groups. FEV_1_% pred, and the FEV_1_/FVC ratio in both groups of women with COPD showed differences when compared with the H-BS, H-TS and C groups (*P < 0.01*) with no difference between the groups with COPD. Women with COPD-TS, COPD-BS, H-TS and H-BS were shorter than the C (*P < 0.01*). The average exposure to BS in the COPD-BS group was 361 ± 177 h/year, while in the COPD-TS group; there was an average cumulative tobacco consumption of 36 ± 23 packs/year.

**Table 1:** Anthropometric, clinical, and physiological characteristics of the study in women. Data are expressed as the mean ± SD (*n*=25)

Group	C	COPD-TS	COPD-BS	H-TS	H-BS
Characteristics					
Age (years)	66.59 ± 8.11	69.4 ± 7.09	73.04 ± 6.66	62.12 ± 13.18	65.54 ± 11.52
Height (cm)	158 ± 8.10	153.92 ± 8.72	147.67 ± 8.45*/*	160 ± 5.9	144 ± 8.60*/*
Weight (Kg)	68.55 ± 11.59	68.8 ± 11.58	58.10 ± 12.25	71.77 ± 19.56	61.62 ± 12.85
BMI (Kg/m^2^)	27.33 ± 3.91	29.21 ± 5.48	26.73 ± 5.62	28.86 ± 5.48	29.63 ± 5.04
*Physiological characteristics*					
FEV_1_ % pred	96.25 ± 3.1	68.81 ± 5.26*	71.95 ± 6.16*/*	86.32 ± 4.2/*	80.14 ± 2.3/*
FEV_1_/FVC ratio	80.02 ± 2.4	58.13 ± 3.1	59.12 ± 4.0	74.12 ± 2.3	74.5 ± 2.2*/*
GOLD grades	Case numbers (%)				
I	0	4 (16)	4 (16)	0	0
II	0	21 (84)	21 (84)	0	0

*Abbreviations*: *BMI* body mass index, *C* control healthy women, *COPD-BS* COPD secondary to biomass smoke exposure, *COPD-TS* COPD secondary to tobacco smoking, *FEV1% pred* forced expiratory volume in 1 second (% predicted), *FVC* forced vital capacity, *H-BS* exposed to BS without COPD, *H-TS* exposed to TS without COPD. Data were analysed by a one-way ANOVA and Tukey's post hoc test. * *P* <0.01; * vs control; /* vs COPD-TS, H-TS or H-BS groups, respectively

### Differential expression of miRNAs in serum by PCR arrays

The analyses of expression of miRNAs were performed on samples from 3 women chosen in a simple random way in each study group. Six miRNAs were differentially expressed; 3 were up-regulation, miR-150-5p, miR-191-5p and miR-223-3p, in the COPD-BS group compared with the H-BS group, and the remaining 3 were down-regulated, miR-374a-5p in the COPD-BS group compared with C, miR-21-5p in the COPD-TS group compared with C, and miR-34a-5p which was down-regulated in the COPD-BS group compared with the COPD-TS group (Table 2).

**Table 2:** miRNA differentially expressed in the serum of women in the COPD-BS, COPD-TS (GOLD stages I–II), H-BS and C groups

Compared groups	microRNA	Regulation level	Fold change	*P*-value
COPD-BS vs. COPD-TS	hsa-miR-34a-5p	down-regulated	−8.77	*0.020460*
COPD-BS vs. C	hsa-miR-374a-5p	down-regulated	−12.29	*0.000291*
COPD-BS vs. H-BS	hsa-miR-150-5p	up-regulated	20.66	*0.000064*
	hsa-miR-191-5p	up-regulated	24.95	*0.004144*
	hsa-miR-223-3p	up-regulated	30.19	*0.000645*
COPD-TS vs. C	hsa-miR-21-5p	down-regulated	−17.89	*0.007987*

Data are expressed as the regulation, fold change and P-value

*Abbreviations*: *C* control healthy women, *COPD-BS* COPD by biomass smoke exposure, *COPD-TS* COPD by tobacco smoke, *H-BS* women exposed to BS without COPD

### Validation of miRNAs by RT-qPCR

**Fig. 2:**
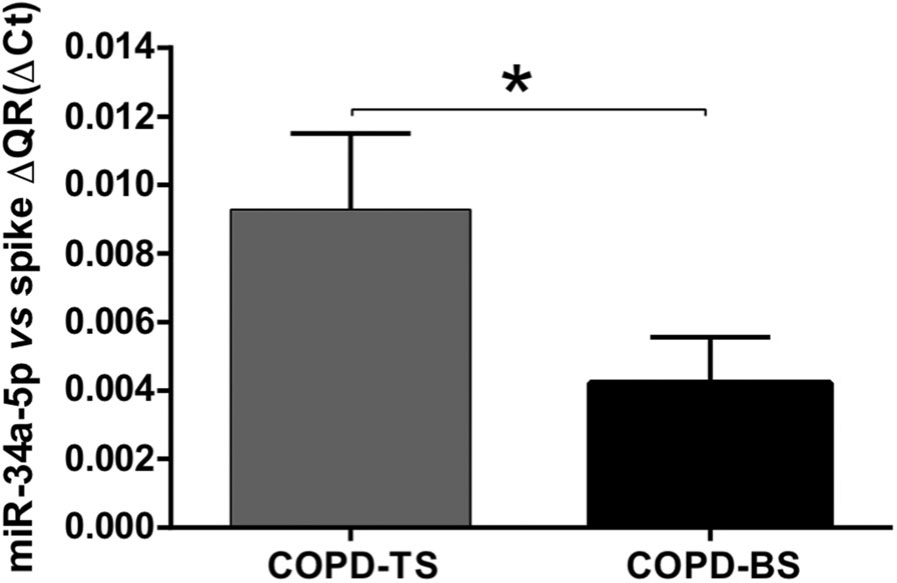
miR-34a-5p is down-regulated in COPD-BS compared COPD-TS. RT-qPCR analysis of serum miR-34a-5p in women with COPD-TS compared with those with COPD-TS and presented as ΔCt values. *n* = 25. A statistical difference was observed between women with COPD-BS and women with COPD-TS. Student’s t-test was used. * *P* < 0.001. Abbreviations: COPD-TS, COPD due to tobacco smoke; COPD-BS, COPD due to biomass smoke.

**Fig. 3:**
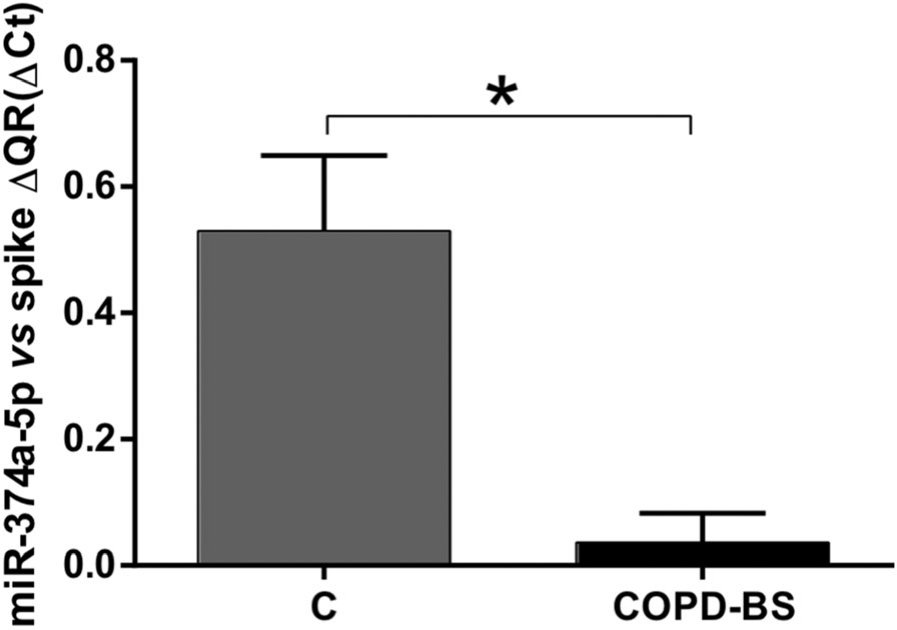
miR-374-5p is down-regulated in COPD-BS compared with C. RT-qPCR analysis of serum miR-374-5p in women with COPD-BS compared with C; *n* = 25. The data are presented as ​ΔCt values. Student’s t-test was used. COPD-BS, COPD due to biomass smoke; C, control women. * *P* < 0.001

**Fig. 4:**
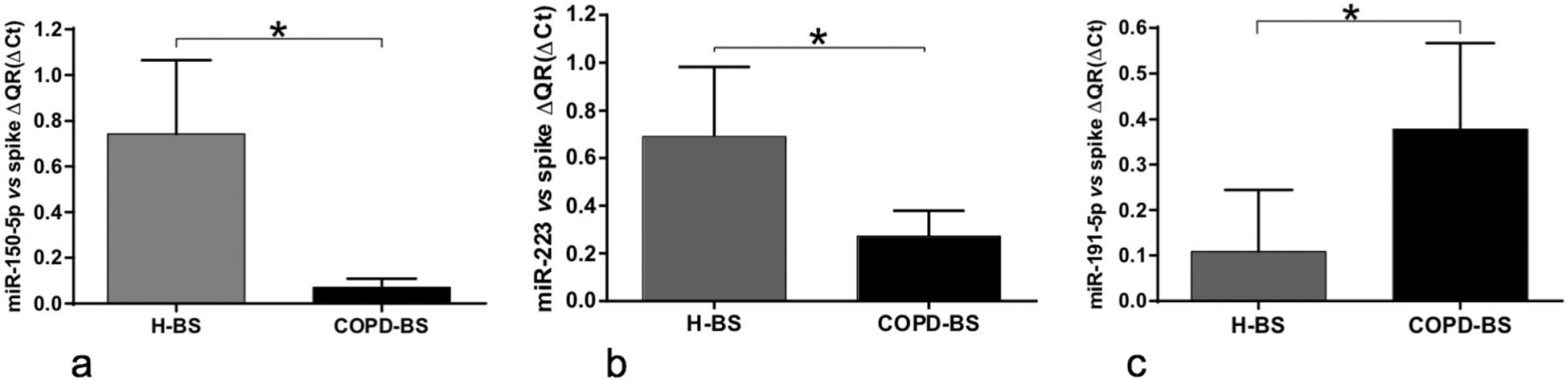
miR-150-5p and miR-223-3p are down-regulated and miR-191-5p is up-regulated in women with COPD-BS compared with H-BS. RT-qPCR analysis of serum miR-150-5p (**a**), miR-223-3p (**b**), and miR-191-5p (**c**) in women with COPD-BS compared with H-BS; n = 25. The data are presented as ΔCt values. Student’s t-test was used. COPD-BS, COPD due to biomass smoke; H-BS, women exposed to BS without COPD. ** *P* < 0.01, * *P* < 0.001

**Fig. 5:**
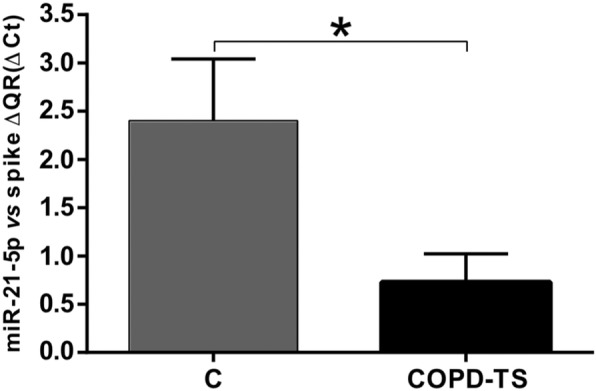
miR-21-5p is down-regulated in women with COPD-TS compared with C. RT-qPCR analysis of serum miR-21-5p in women with COPD-TS compared with C; *n* = 25. The data are presented as ΔCt values. ​ Student’s t-test was used. COPD-TS, COPD due to tobacco smoke; C, control women. *** *P* < 0.05

To validate the differentially expressed miRNAs obtained in the PCR matrices (*n* = 3), the cDNAs were obtained using the RT kit and TaqMan Universal Master Mix II with UNG (Applied Biosystems-Thermo Fisher Scientific). The six miRNAs validated by RT-qPCR (*n* = 25) were as follows: miRNA-34a-5p was down-regulated in the COPD-BS compared with the COPD-TS group (Fig. 2; *P* < 0.001), miR-374a-5p was down-regulated in the COPD-BS compared with the controls (Fig. 3; *P* < 0.001), miR-150-5p that was down-regulated in the PCR array analysis did not correspond with the study being decreased by RT-qPCR (Fig. 4a; *P* < 0.01). The same result was observed with miR-223-3p (Fig. 4b; *P* < 0.001), when comparing women from the COPD-BS group with those from the H-BS group, while miR-191-5p corresponded with the PCR result and was up-regulated in the COPD-BS group compared with the H-BS group (Fig. 4c; *P* < 0.01), and miR-21-5p was down-regulated in the COPD-TS group compared with the controls (Fig. 5; *P* < 0.05).

### Serum Notch1 concentration

To interpret the possible biological relevance of the detected miRNAs in the pathogenesis of COPD, an analysis of the targets of miR-34a-5p was performed specifically because of the subexpression observed in the COPD-BS group compared with the COPD-TS group.

**Fig. 6:**
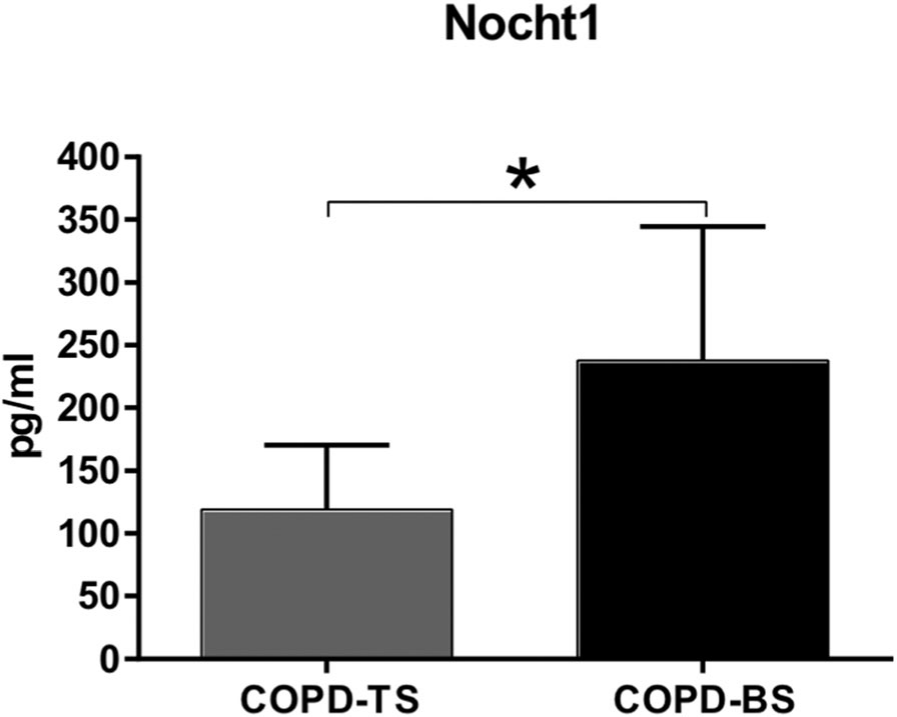
serum Notch1 is higher in COPD-BS than in COPD-TS. The protein Notch1 was quantified in serum by ELISA and expressed in pg/ml. A statistical difference was observed between women with COPD-BS and women with COPD-TS. *n* = 25. Student’s t-test was used. * *P* < 0.001. Abbreviations: COPD-TS, COPD due to tobacco smoke; COPD-BS, COPD due to biomass smoke

The target was searched in the updated database DIANA TOOLS, miRTarBase-bio.tools and TargetScan. The investigated focused on the participation of miR-34a in COPD-TS and other pulmonary diseases resulting in the Notch1 protein. The serum concentration of Notch1 was quantified by ELISA and was elevated in women in the COPD-BS group compared with women in the COPD-TS group (Fig. 6; *P* < 0.001) and exhibited an inverse association with the expression of miR-34a-5p.

## Discussion

We aimed to analyze the differential expression of miRNAs across five groups of participants with and without COPD by tobacco and biomass exposure. The main finding was that miR-34a down-regulated was differentially expressed between COPD-TS and COPD-BS. We detected differences in 5 miRNAs, miR-374a miR-191-5p, miR-21-5p, miR-150, miR-223, yet, these differences were not statistically different between COPD-TS and COPD-BS.

A differential expression of miR-34 in TS-COPD compared to healthy subjects has been reported; however, in the TS-COPD case miR-34 was up-regulated, with a consequent activation of p53. The expression of miR-34 has been also linked to the severity of TS-COPD, suggesting that miR-34a contributes to the pathogenesis of COPD, by activation in the HIF-1α pathway (hypoxia-inducible factor) [14]. Another study reported that miR-34a activation is induced by oxidative stress through PI3K (phosphoinositide-3-kinase) signaling, and it is implicated in aging responses to oxidative stress; thus, miR-34a could become a new therapeutic target and biomarker in COPD and age-related diseases driven by oxidative stress [15]. Contrary to the up-regulated of miR34a in COPD-TS, our results in COPD-BS are down-regulated, which probably gives us the preliminary basis for inferring that miR-34a could distinguish the genotypic characteristics of COPD-BS patients with respect to COPD-TS patients.

To understand one of the possible biological implications of the down-regulation of miR-34a in COPD-BS, one of its targets, the Notch1, was selected. miR-34a reduces the action of the Notch1 pathway, which plays an important role in the differentiation of the epithelium in the human airway. It has been observed that the addition of a Notch1 ligand, or the constitutive expression of its receptor, increases the number of mucosal cells containing MUC5AC and the number of secretory cells [16, 17]. Focusing on our findings, we can infer that patients with COPD by biomass, which have miR-34a down-regulated, do not supress the activation of Notch1 signaling, increasing the number of secretory cells, as has been shown in in vitro studies [17]. Our finding provides a potential explanation for the chronic bronchitis clinical expression of COPD-BS, likely mediated by the down-regulation of miR-34a. This is relevant, because the regulation of Notch 1 could represent an important therapeutic target for these patients.

Another interesting finding of our study is the differential expression of three miRNAs between the study groups: miR-374a down-regulated in the group C vs COPD-BS, miR-191 up-regulated in the group H vs COPD-BS and miR-21 down-regulated in the group C vs COPD-TS. The miR-374a has been reported to be up-regulated in skeletal muscle in patients with COPD-TS, and associated with the development of extrapulmonary manifestations and co-morbidities in COPD-TS [18]. Base on the findings we suggest that it could be a good indicator of the comorbidities of COPD-BS patients, although more in-depth studies are needed to determine this possibility. miR-191, has been reported to be up-regulated in lung tissue and bronchoalveolar lavage (BAL) of mice exposed to TS, associated with the development of inflammatory cells in lung and lung parenchyma [19]. The results suggest that COPD due to tobacco and biomass could share the same pathway of inflammation; however, our results need to be evaluated with more studies. Another miRNA validated was miR-21, this miR in COPD-TS has been reported as up-regulated in asymptomatic smokers [20]. Another study demonstrated that up-regulated of miR-21 in plasma and mononuclear cells of patients with COPD-TS may contribute to their pathogenesis and severity [21], suggesting that high serum plasma levels of miR-21 may be a diagnostic and therapeutic indicator in COPD-TS [20], so that, our results were consistent with previous studies.

### Limitations of the study

The limitations of this study are related to its sample size. We used a small number of participants in the screening phase, to identify key miRNAs to be later validated in the total sample. This procedure will lead to the identification of miRNAs that are very different across groups but will fail to identify miRNAs that are more similar, which could cloud our understanding of partially expressed miRNAs. For this purpose, a larger sample size is needed. Additionally, only Notch1 was quantified, one of the many targets of miR-34a. Still, this limited analysis allowed us to begin to understand the relevance of miRNAs in COPD-BS, considering the great complexity of this disease. Coupled with this, characteristics such as the socio-economic level, the level of education, ethnic origin, genetic susceptibility and various other environmental factors, as well as the type and severity of exposure to BS or TS in the study groups could influence our results.

## Conclusions

This is the first study in patients with COPD due to biomass that demonstrates the genotypic difference between patients determined by miRNAs, supporting that COPD by biomass has a different genotype than COPD due to tobacco, which could have important implications for the treatment of these diseases.

## Data Availability

The datasets generated during and/or analyzed during the current study are available from the corresponding author on reasonable request.

## Abbreviations

BMI: Body mass index
BS: Biomass smoke exposure
C: Control healthy women
COPD-BS: COPD by biomass smoke exposure
COPD-TS: COPD by tobacco smoking
FEV_1_% pred: forced expiratory volume in the 1st sec (% predicted)
FVC% pred: forced vital capacity (% predicted)
GOLD: Global Initiative for Chronic Obstructive Lung Disease
H-BS: Women exposed to BS without COPD
H-TS: Smokers without COPD
miR: microRNA
miRNAs: MicroRNAs
RT-qPCR: Real-time reverse transcriptionase-PCR
TS: Tobacco smoking
